# Artemisinin resistance in rodent malaria - mutation in the AP2 adaptor μ-chain suggests involvement of endocytosis and membrane protein trafficking

**DOI:** 10.1186/1475-2875-12-118

**Published:** 2013-04-05

**Authors:** Gisela Henriques, Axel Martinelli, Louise Rodrigues, Katarzyna Modrzynska, Richard Fawcett, Douglas R Houston, Sofia T Borges, Umberto d’Alessandro, Halidou Tinto, Corine Karema, Paul Hunt, Pedro Cravo

**Affiliations:** 1 Centro de Malaria & Doenças Tropicais.LA/IHMT/Universidade Nova de Lisboa, Lisbon, Portugal; 2 Institute for Immunology and Infection Research, School of Biological Sciences, University of Edinburgh, Edinburgh, UK; 3 Institute for Structural and Molecular Biology, School of Biological Sciences, University of Edinburgh, Edinburgh, UK; 4 Prince Leopold Institute of Tropical Medicine, Antwerp, Belgium; 5 Centre Muraz/Institut de Recherche en Sciences de la Santé, Bobo Dioulasso, Burkina Faso; 6 National Malaria Control Programme, Kigali, Rwanda; 7 Centre of Immunity, Infection and Evolution, School of Biological Sciences, University of Edinburgh, Edinburgh, UK; 8 Instituto de Patologia Tropical e Saúde Pública/Universidade Federal de Goiás, CAPES/Brazil, Goiânia, 74605-050, Brazil; 9 Current address: London School of Hygiene and Tropical Medicine, Room 490, Keppel Street, London, WC1E 7HT, UK; 10 Current address: Wellcome Trust Sanger Institute, Hinxton, Cambridgshire, UK

**Keywords:** Malaria, Artemisinin, Drug resistance, Genomics, Plasmodium chabaudi, Mutation, Endocytic machinery

## Abstract

**Background:**

The control of malaria, caused by *Plasmodium falciparum,* is hampered by the relentless evolution of drug resistance. Because artemisinin derivatives are now used in the most effective anti-malarial therapy, resistance to artemisinin would be catastrophic. Indeed, studies suggest that artemisinin resistance has already appeared in natural infections. Understanding the mechanisms of resistance would help to prolong the effective lifetime of these drugs. Genetic markers of resistance are therefore required urgently. Previously, a mutation in a de-ubiquitinating enzyme was shown to confer artemisinin resistance in the rodent malaria parasite *Plasmodium chabaudi.*

**Methods:**

Here, for a mutant *P. chabaudi* malaria parasite and its immediate progenitor, the *in vivo* artemisinin resistance phenotypes and the mutations arising using Illumina whole-genome re-sequencing were compared.

**Results:**

An increased artemisinin resistance phenotype is accompanied by one non-synonymous substitution. The mutated gene encodes the μ-chain of the AP2 adaptor complex, a component of the endocytic machinery. Homology models indicate that the mutated residue interacts with a cargo recognition sequence. In natural infections of the human malaria parasite *P. falciparum,* 12 polymorphisms (nine SNPs and three indels) were identified in the orthologous gene.

**Conclusion:**

An increased artemisinin-resistant phenotype occurs along with a mutation in a functional element of the AP2 adaptor protein complex. This suggests that endocytosis and trafficking of membrane proteins may be involved, generating new insights into possible mechanisms of resistance. The genotypes of this adaptor protein can be evaluated for its role in artemisinin responses in human infections of *P. falciparum*.

## Background

Without an effective vaccine, prevention and treatment of human malaria has traditionally relied on chemoprophylaxis and/or chemotherapy [1]. However, *Plasmodium falciparum* has developed resistance to nearly every anti-malarial drug introduced to date, compromising its control. Resistance arises via the selection of parasites bearing specific mutations, and is decisive in determining the effective lifetime of anti-malarial agents.

Artemisinin combination therapy (ACT) is now a widely used anti-malarial treatment. The artemisinin component is a highly effective and rapidly acting drug [2]. The understanding of its mode of action is incomplete [3,4], but independent lines of evidence from a number of laboratories suggest that its action depends upon its endoperoxide group and activation by haem or other iron sources [3]. Downstream, it may localize close to the digestive vacuole (DV) [5] and effect changes in DV morphology [6] or the distribution, endocytosis and digestion of haemoglobin [7]. Alternatively, it has been suggested that artemisinin may inhibit the Ca^2+^-ATPase (PfATP6) [8].

Alarmingly, recent data indicate that resistance to artesunate, one of several artemisinin derivatives (ARTDs), is emerging in Cambodia and Thailand [9-14]. Molecular (DNA) markers of resistance are therefore required urgently. These tools will help to monitor the evolution of resistance, to establish rational treatment policies and to design drug combinations that delay the evolution of resistance. At present however, in *P. falciparum*, there are no universally accepted and validated molecular markers of artemisinin resistance in the field. Polymorphisms in PfATPase6 [15,16] or amplification of the multidrug resistance gene, *Pfmdr1*[17-19] have been investigated. However, no correlation was found between variants of these genes and *in vivo* responses to ARTDs in the first suggested cases of resistance along the Thai/Cambodian border [20,21].

Candidate gene approaches may not focus on the critical genes nor grasp the full complexity of the drug response mechanisms. Instead, analysis of genetic haplotype variation and conservation and geographic differentiation using genome-wide SNP typing of *P. falciparum* parasites from Southeast Asia (Thailand, Cambodia and Laos), where resistance to ARTDs is emerging, has identified candidate regions associated with slow parasite clearance rates after drug treatment [21], particularly in a region on chromosome 13. No specific genes in this region were identified as candidates for influencing artemisinin response.

Other experimental studies that do not prejudge the critical genes are especially informative; for example, the *in vitro* generation of mutant *P. falciparum* parasites resistant to drugs. Parasites resistant to ARTs have been generated and *mdr1* duplications identified [22], but often the phenotypes and genetic changes have tended to be unstable in the absence of drugs [23,24]. Genetic linkage analysis of the experimental *P. falciparum* Hb3 x Dd2 cross has shown that three *loci*, including *pfmdr1* and two additional *loci* (on chromosomes 12 and 13) [24] that were associated with artemisinin (ART) responses or the potential to evolve ART resistance, but neither of these parental parasites offered a distinct ART-R phenotype.

**Figure 1:**
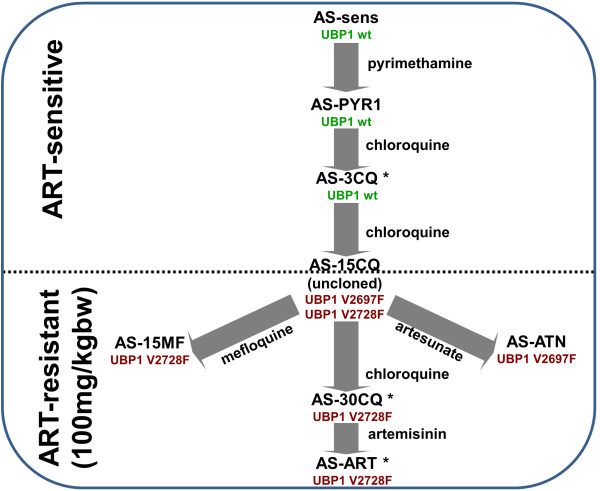
**The AS lineage of drug resistant parasites, featuring artemisinin (ART) phenotypes and *ubp-1 *genotypes.** Parasites were selected by passage in presence of drugs shown [29-33]. Artemisinin resistance (phenotype 1) appears during selection by chloroquine [25,26] and is mediated by either of two mutations in the *ubp-1* gene (PCHAS_020720) [25,27]. AS-ART was generated during selection by artemisinin [29]. Its increased resistance to artemisinin (phenotype 2) and its genotype is the subject of the present study. *, parasites used in the present study; wt, wild-type; kg bw, kilograms body weight.

*In vivo* experimental studies using the rodent malaria *Plasmodium chabaudi* can circumvent some limitations faced by *in vitro* experimentation. A lineage (strain AS) of genetically stable mutant parasite clones that are resistant to various drugs has been generated by experimental evolution under drug selection (Figure 1). The genetic mutations conferring resistance to pyrimethamine, sulphadoxine, chloroquine, mefloquine, lumefantrine and artemisinin have been mapped using efficient genetic linkage mapping (population based genome-wide scans of selection) and Illumina whole-genome sequencing [25-28]. The identification of the critical mutations exploits four important features relevant to the present study. Firstly, the resistance phenotype appears at the same time/position in the lineage as the critical mutation. Secondly, the mutation will lie at the bottom of a ‘selection valley’ (genomic region selected by drug). Thirdly, these studies exploited a previously completed reference genome sequence isogenic to the progenitor parasite, AS-sens. Fourthly, the numbers of point mutations (genome-wide) fixed at each step by selection or during cloning are very small, typically 1–3 [26].

For artemisinin, a mutation in a de-ubiquitinating enzyme (V2728F *ubp1*) was proposed previously as the critical determinant of an artemisinin-resistance (ART-R) phenotype [25] in parasite AS-30CQ (Figure 1). This *ubp1* mutation has also been shown to contribute to resistance to mefloquine and lumefantrine [27] and higher doses of chloroquine [26], as well as ARTs, suggesting that *ubp1* may mediate parasite responses to multiple drugs, as does *mdr1* duplication [27].

Here, another parasite, AS-ART (Figure 1), derived from AS-30CQ after prolonged and progressive ART selection [29], is investigated. An increased ART-R phenotype is characterized and a single point mutation in its genome is defined. The 3D structures of homologues of the mutated protein are investigated for possible functional consequences. Allelic variations of the *P. falciparum* orthologue of the mutated gene are defined in a set of *P. falciparum* field samples.

## Methods

### Ethics

All animal work was conducted according to relevant national and international guidelines: in Portugal, after approval by the Ethics Committee of the Instituto de Higiene e Medicina Tropical of Lisbon, Portugal, under PARECER 2/2006 from 1 August, 2006 and in the UK, in compliance with the UK Animals (Scientific Procedures) Act 1986.

### Parasite lines, maintenance, parasite preparation and DNA extraction

All the parasite clones used in this study are members of the AS lineage (Figure 1). Three parasite clones were used: i) AS-3CQ, a parasite that is resistant to low levels of chloroquine [34]; ii) AS-30CQ, that is resistant to high levels of chloroquine [34]; and, iii) AS-ART, which was derived from AS-30CQ through multiple sub-inoculations in mice under increasing doses of artemisinin (ART) [29]. All parasites were routinely inoculated, passaged in CBA mice (four to six weeks) and cryopreserved as previously described [30]. Parasites were prepared and DNA extracted as previously described [35], ensuring that host white cells were removed by CF11 cellulose (Whatman) and Plasmodipur filters (Eurodiagnostica). DNA samples from other clones within the lineage were used to determine the earliest appearance of specific mutations within the lineage. All animal work was conducted according to relevant national and international guidelines: in Portugal, after approval by the Ethics Committee of the Instituto de Higiene e Medicina Tropical of Lisbon, Portugal, under PARECER 2/2006 from August 1st 2006 and in the UK, in compliance with the UK Animals (Scientific Procedures) Act 1986.

### *In vivo* ART drug tests

*In vivo* ART response phenotypes were assessed as follows. Four to six-week-old inbred CBA mice were divided into nine groups of three mice each. Mice in groups 1–3 were inoculated with 10exp6 parasitized red blood cells (pRBC) of AS-3CQ, AS-30CQ or AS-ART, respectively and given the diluting solvent DMSO orally (untreated controls). Treatment groups 4–6 were inoculated with 10exp6 pRBC of either AS-3CQ, AS-30CQ or AS-ART and treated with an oral daily dose of 200 mg ART kg^-1^ mouse bodyweight, administered for a total of three consecutive days, starting day 1 post-inoculum (pi). Treatment groups 7–9 were inoculated with 10exp7 pRBC of either AS-3CQ, AS-30CQ or AS-ART and treated with 200 mg ART kg^-1^ mouse bodyweight, administered for a total of five consecutive days. Individual percentage (%) parasitaemia was followed from day 4 pi onwards and up to day 15 in the case of untreated control mice. For treated mice, % parasitaemia was first assessed one day after the last day of treatment and up to day 18 pi. Results were expressed as daily average % parasitaemia ± standard error from the three mice within each experimental group.

### Genome-wide resequencing

Clone AS-sens was previously resequenced with the Illumina® platform using 36 bp single reads [25]. Clone AS-ART was also re-sequenced using 36 bp single-end reads. Individual sequence strings (reads) from AS-sens and AS-ART were aligned against the isogenic AS reference genome assembly (AS-WTSI [36], provided by the Wellcome Trust Sanger Institute, using two different software packages: MAQ (Mapping and Assembly with Quality) [37] and SSAHA2 (Sequence Search and Alignment by Hashing Algorithm) [38]. The AS-WTSI sequence data consisted of a recently completed assembly and annotation made available during this investigation. Detection of SNPs was performed with Samtools [39] and MAQ, using default parameters, as described in Hunt *et al.*[25]. Small (≤3 bp) indels were detected using Samtools internal algorithm only. The list of single nucleotide polymorphisms (SNPs) and small indels were further filtered by removing mutations proposed for both AS-sens and AS-ART and that therefore did not arise within the AS lineage. Heterozygous and “multiple variant” SNPs, as well as small indels called by less than three reads and less than 50% of the total reads were also removed. Larger indels (>3 bp) and CNVs were detected with both MAQ and SSAHA2 using “comparative coverage” analysis, which measures the ratio of the relative coverage (local coverage divided by overall mean coverage) in AS-ART relative to AS-sens. A comparative coverage ratio >1.5 over 200 bp for defining CNVs and <0.25 over 10 bp for defining indels (both adjusted for different genome coverage in AS-sens and AS-ART) were used, as previously described [25]. Unlike previous work, “comparative coverage” analysis was also performed using MAQ. This required running the “pileup” command on both the AS-ART and AS-sens reads using the variable “-q 1” (which excludes reads with a mapping quality <1). The pileup files thus produced were then used for “comparative coverage” analysis with custom made scripts, similarly to the SSAHA2 approach. Confidence levels were assigned to mutations based on the following criteria: a) Samtools quality scores (for SNPs) and b) identification by both MAQ and Samtools (for SNPs and large indels/CNVs). All high confidence putative point mutations proposed by MAQ and/or Samtools were verified by di-deoxy sequencing. Only limited verification was done for potential indels and CNVs. Small indels were defined as “low confidence” mutations by default, due to a majority of calls being confirmed as false positives by di-deoxy sequencing in this and other studies [25,28].

### Protein structure-function analysis

The *P. chabaudi* AP2-μ sequence was used to search the protein structure database (PDB) for homologues. The closest match found was the μ chain of AP2 from *Rattus norvegicus*, with a bit score of 167 and an *E*-value of 2e-41. 11 different structure entries of this and related proteins and complexes were present in the PDB. Structure 2PR9 was selected because of the level of R-factors (R: 0.204, R-free: 0.240) and its resolution (2.51Å).

The I-TASSER server [40] was used to construct a homology model of the *P. chabaudi* homologue, with the *R. norvegicus* 2PR9 structure as a template onto which the *P. chabaudi* sequence was modelled. The same method was used to construct a *P. falciparum* μ model.

### *Plasmodium falciparum ap2-μ* genotyping

In order to investigate the genetic variation in the *P. falciparum ap2-μ* gene in natural infections, DNA was extracted from field samples collected in Africa and South America, as well as from the reference strain 3D7, according to the method described by Plowe and co-workers [41]. The *P. falciparum ap2-μ* gene was thus sequenced in a subset of 24 *P. falciparum* isolates collected within the scope of previous studies, of which eight were from Rwanda [42], eight from São Tomé [43] and eight from Brazil [44].

The sequence of the *P. falciparum* orthologue of the *ap2-μ* gene (accession no. PF3D7_1218300) was retrieved from PlasmoDB and used as template for designing primers to amplify its open reading frame using an overlapping PCR fragment strategy (see Additional file 1). PCR assays were performed with 1 μl of DNA into a 50 μl mixture containing 0.2 μM of each primer, 1.5 mM MgCl2, 0.2 mM deoxynucleoside triphosphates and 1.25 units of GoTaq® Flexi DNA Polymerase (Promega).

PCR products were analysed by ethidium bromide-stained agarose 2% gel electrophoresis and sequenced directly in both sense and antisense directions with the appropriate PCR amplification primers.

## Results

### The *Plasmodium chabaudi* AS-ART phenotype

Here, the ART responses of isogenic parasites of the *P. chabaudi* AS lineage are investigated further; namely, AS-3CQ (ART-S clone), AS-30CQ (ART-R clone) and the AS-ART (ART-R clone) generated during selection by ART (Figure 1) [29]. In previous work, artemisinin resistance at 100 mg ART kg^-1^ (body weight) d^-1^ was demonstrated in both AS-30CQ and AS-ART relative to other clones, which preceded them in the AS lineage, including AS-3CQ [25]. Here, the hypothesis that AS-ART would resist higher doses of ART than AS-30CQ is tested. Parasites were treated for three (1 × 10 exp6 inoculum) or five (1 × 10 exp7 inoculum) days (treatments A and B, respectively) with a daily dose of 200 mg ART kg^-1^ d^-1^ and their response to the drug was assessed by comparing both peak parasitaemia and the time taken for each parasite to recrudesce after treatment.

**Figure 2:**
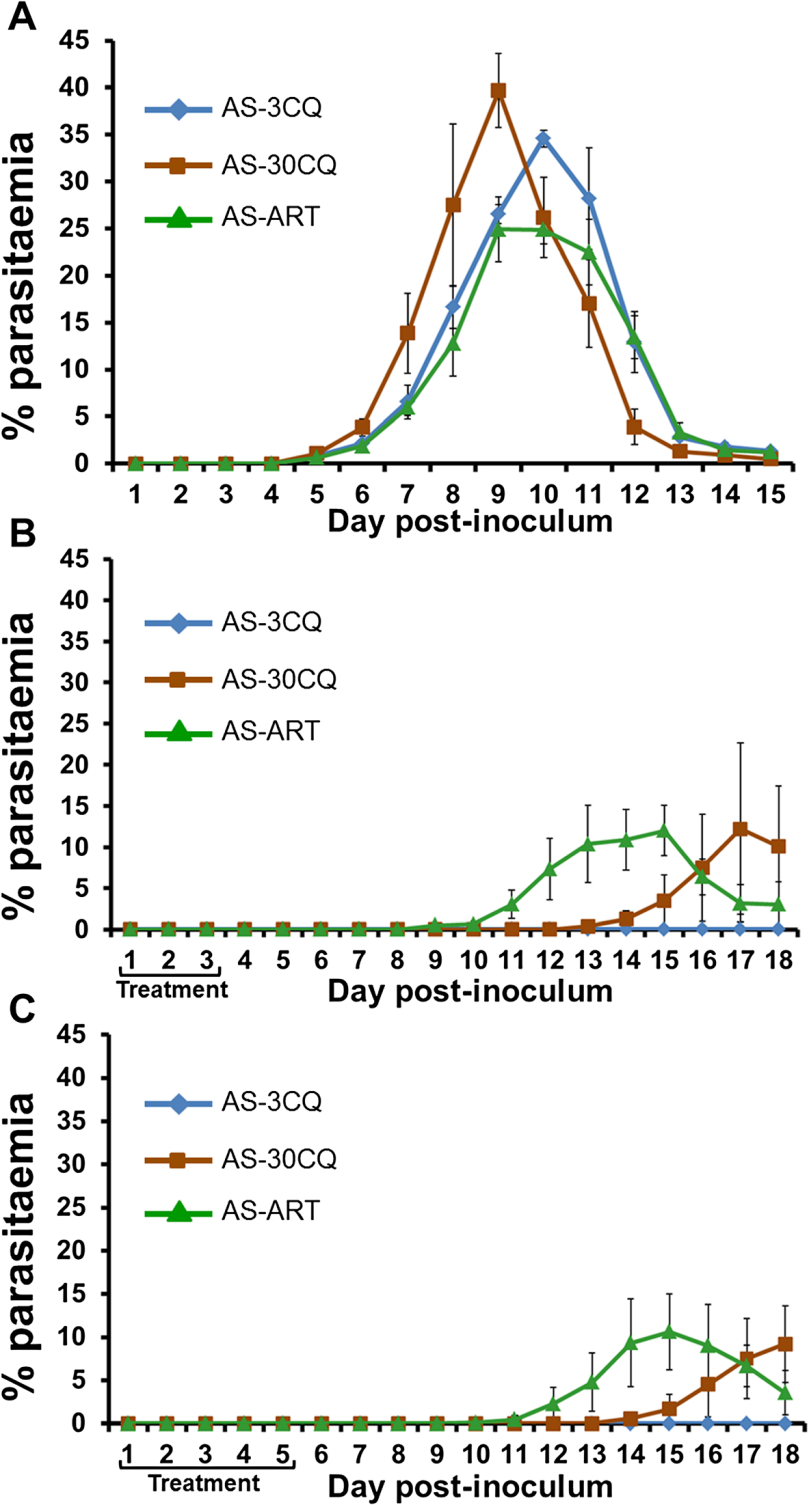
**Artemisinin responses of *Plasmodium chabaudi *AS-3CQ, AS-30CQ and AS-ART.** Mean% parasitaemia ± standard error of groups of three mice. **A**, untreated controls; **B**, 1 × 10exp6 parasites on day 0 and treated with 200mg ART/kg^-1^ day^-1^ days 1–3; **C**, 1 × 10exp7 parasites on day 0 and treated with 200mg ART/ kg^-1^ day^-1^ days 1–5. AS-sens, blue; AS-30CQ, red; AS-ART, green.

In the absence of treatment, all three parasite clones produced their peak parasitaemia of between 23% (AS-ART) and 40% (AS-30CQ), appearing between days 8 and 9 pi (Figure 2A). Under either ART treatment A and B however, no parasites were detected in mice infected with AS-3CQ over the follow-up period (Figure 2B and 2C). After treatment A, AS-30CQ produced detectable parasites between days 13–14, with parasitaemia reaching their peak (~12%) on day 17. However, mice infected with AS-ART presented more rapid recrudescence (days 8–9), with parasitaemia reaching ~12% on day 15 after treatment (Figure 2B). A similar trend was observed after treatment B: mice infected with AS-30CQ first presented a detectable parasitaemia between days 13–14 and peak parasitaemia (~10% on day 18), whilst the AS-ART group recrudesced three days earlier (Figure 2C) achieving a similar peak parasitaemia on day 15.

As previously reported [25] it is concluded that AS-3CQ is sensitive to ART treatment whereas both AS-30CQ and AS-ART are resistant. Here, however, at high ART doses, AS-ART shows both earlier recrudescence and reduced time to reach peak parasitaemia relative to AS-30CQ. It is concluded that AS-ART has a higher degree of resistance to ART. Here, this enhanced resistance phenotype is called ART-R phenotype 2. The response of AS-30CQ and AS-ART to 100 mg ART kg^-1^ d^-1^ (3 day) is called ART-R phenotype 1.

### Genome re-sequencing of AS-ART

AS-ART was sequenced using the Illumina platform using 36 base single-end reads. In total, 45,879,892 short single-end reads were produced for clone AS-ART, of which 89% were mapped onto the 2009 version of the reference genome (Welcome Trust Sanger Institute *P. chabaudi* AS parasite, AS-WTSI, genome size 18,832,196 bp [36]: by the mapping software, SSAHA2. The mean genome-wide read coverage was 84 reads per nucleotide (see Additional file 2). Approximately 87% of the total reads (39,979,351) were mapped in unique positions. Some 98% of the nucleotides in the genome were covered by at least 10 reads and 93% by at least 40 reads; 1.67% was covered by less than three reads (the minimum required for SNP and small indel detection). Similar data were obtained using an alternative mapping software, MAQ. Clones AS-sens and AS-30CQ had been re-sequenced previously and reads aligned similarly [25,26,28].

### SNP detection

Preliminary SNP calling and filtering (see Methods) identified eight point mutations in AS-ART (Table 1), relative to the resequenced clone AS-sens [25-28]. There were 11 additional low- quality SNP calls considered to be false positives (see Additional file 3).

**Table 1:** **Confirmed and high confidence mutations in the ART-resistant *Plasmodium chabaudi *clone AS-ART (relative to progenitor clone, AS-sens)**

**Chromosome**	**Type**	**Analysis**	**Nucleotide start**	**Nucleotide end**	**reference base**	**variant base**	**SSAHA quality**	**Confirmation of mutation by dideoxy sequencing**	***P. chabaudi *gene ID**	**Amino acid change**	**Nearest gene ID ( *P. chabaudi *)**	***P. falciparum *orthologue**
**SNPs**												
2	SNP	SSAHA/MAQ	216,954		C	A	99	confirmed*	PCHAS_020720	**V2728F**		PF3D7_0104300
3	SNP	SSAHA/MAQ	70,553		G	T	99	confirmed*	PCHAS_030200	**T707N**		None
3	SNP	SSAHA/MAQ	474,123		C	A	99	confirmed*	PCHAS_031370	**T719N**		PF3D7_0214800
7	SNP	SSAHA/MAQ	994,546		G	A	99	confirmed*	PCHAS_072830	**S106N**		PF3D7_0417200
10	SNP	SSAHA/MAQ	634,932		T	C	99	confirmed*	PCHAS_101550	**Y162H**		PF3D7_1430000
11	SNP	SSAHA/MAQ	996,332		G	T	99	confirmed*	PCHAS_112780	**A173E**		PF3D7_0629500
14	SNP	SSAHA/MAQ	936,945		T	G	92	confirmed*			5'-PCHAS_142600	PF3D7_0813400
14	SNP	SSAHA/MAQ	1,270,184		T	C	99	confirmed*	PCHAS_143590	**I568T**		PF3D7_1218300
					**approximate size**		**comparative coverage**					
**Indels**												
4	indel	SSAHA/MAQ	793,940	793,988	49		0.16	tbc**			PCHAS_042080-5	None
5	deletion	SSAHA/MAQ	683,724	684,989	1,266		0.21	tbc**	PCHAS_051920			None
7	deletion	SSAHA/MAQ	876,907	876,921	15		0.18	34bp deletion*			PCHAS_072420-3'	PF3D7_0815700
bin	indel	SSAHA/MAQ	261,129	284,496	23,368		0.02	tbc**	PCHAS_000700-760		None	

All point mutations and 34 bp deletion (*) were confirmed by di-deoxy sequencing. A small deletion on chromosome 4 and large deletions on chromosome 5 and ‘bin’ (**), have strong supporting evidence (extended area of low and zero coverage, SNP-proxies for deletions). Base quality scores (which only apply to SNPs) are derived from the SSAHA2 algorithm (with 99 being the best score). For indels, the approximate size of the affected area (as measured by SSAHA2) and a comparative coverage are given. Amino acid substitutions (non-synonymous mutations) are in bold characters. One point mutation in AP2-μ chain specific to AS-ART (relative to preceding clone, AS-30CQ) is in bold and underline characteres. Tbc: to be confirmed.

Eight point mutations identified by both Samtools and MAQ had higher quality scores (≥92, maximum 99) (Methods) than the other 11 (quality scores <34, see Additional file 3) calls, and were subsequently verified by di-deoxy sequencing in AS-ART (Table 1). Seven of these eight were previously reported from the AS-ART progenitor, AS-30CQ [25,26]. They include five point mutations, previously linked to drug resistance phenotypes. These are: a) S106N *dhfr* (encoding dihydrofolate reductase) (PCHAS_072830), (quality score: 99), which confers pyrimethamine resistance [28]; b) A173E (quality score: 99) in an amino acid transporter (*aat1*, PCHAS_112780), conferring chloroquine resistance [26]; c) T719N (quality score: 99) in a hypothetical protein (PCHAS_031370) conferring intermediate chloroquine resistance [26]; and, d) a V2728F (quality score: 99) substitution in the *ubp1* gene (PCHAS_020720), conferring artemisinin resistance phenotype 1 [25], high-level chloroquine resistance [26] and mefloquine resistance [27]. A fifth point mutation namely T707N (quality score 99) in a member of the *P. chabaudi*-specific interspersed repeat (*cir*) gene family (PCHAS_030200) may also contribute to the intermediate CQ-R phenotype of AS-30CQ [26]. In all cases, the position of their appearance in the *P. chabaudi* AS lineage coincides with the appearance of the corresponding drug-resistance phenotype.

Two point mutations have not been linked to any particular phenotype. These are: Y162H (quality score 99) in a gene annotated as a hypothetical protein (PCHAS_101550) appearing first in AS-30CQ, and an intergenic mutation (quality score: 92) at base position 936,945 on chromosome 14, located between genes PCHAS_142590 and PCHAS_142600 (both hypothetical proteins with no significant Pfam matches), first appearing in AS-PYR1, the first drug-resistance clone of the lineage.

One point mutation *only* in clone AS-ART relative to AS-30CQ was confirmed. This mutation was I568T (quality score 99) in a gene (PCHAS_143590) predicted to encode the mu (μ) chain of the AP2 adaptor protein complex, which, in other organisms, is involved with clathrin-mediated endocytosis [45]. This gene was denoted *ap2*-*μ*.

In contrast to the eight confirmed mutations above, the other 11 low-quality calls (see Additional file 3, highlighted in orange) were predicted only by Samtools; nine of these have extremely low Samtools quality scores (2 to 13) and were variously mapped to the extreme ends of chrs 01, 03, 07 (two), 10 and 12, where *P. chabaudi*-specific genes are located (often in multigene families), or to contigs yet to be mapped to the final genome assembly (three in ‘bin’). Here, read alignment may be less reliable, as reflected in their low mapping quality. A similar low quality SNP call in AS-30CQ was previously identified as a false positive by di-deoxy-sequencing [28]. Accordingly, these nine low confidence SNPs are strongly predicted to be false positives. In any case, their genomic locations suggest that they would be unlikely to play a role in a conserved artemisinin resistance phenotype because four of these candidates were intergenic (chr01, 03, 07, 10) and the other five (chr07, 12 and bin (three)) were in genes without orthologues in *P. falciparum*, in contrast to the eight confirmed point mutations.

The two remaining low confidence point mutations mapped to chr05 or contig11844 and had higher quality scores (33 and 31, respectively). However, they were located close to, or within extended regions of low coverage proposed to be deletions (one of which, on chr05, was previously noted) [26], see below. False positive SNPs such as these were previously termed ‘proxies for deletions’ and their artefactual appearance explained [25].

It is concluded that the I568T mutation in the gene PCHAS_143590 encoding the mu (μ) chain of the AP2 adaptor protein complex is likely to be the only point mutation in AS-ART relative to its immediate precursor AS-30CQ.

### Small indels, larger deletions and CNV detection

Forty-one potential insertions or deletions (indels) (14 larger (>3bp) indels, 27 small (≤3 bp) indels) and seven potential copy number variants (CNVs) were called (see Additional files 4, Additional file 5 and Additional file 6) in AS-ART relative to AS-sens. The specific detection and identification of large insertions was not feasible because the single-end sequencing data used in these studies were not able to support the use of more sophisticated detection algorithms that depend upon the availability of paired-end data.

The 27 small indels were considered to be low confidence predictions, *ie*, false positives. Dideoxy-sequencing confirmed that the five tested were all false positives (see Additional file 4), as in previous studies of AS-15MF and AS-30CQ [25,28]. It is suggested that the majority of these predicted low confidence mutations are therefore false positives. In any case, only six of the other 22 small indels were intragenic.

For larger indels, analysis based on read depth reveals large deletions (say >20 bp) with greater reliability than for intermediate deletions (say 3<bp<20) and all larger insertions. Four (see Additional file 5, highlighted in yellow) of the 14 intermediate and large indels candidates were classified as high confidence mutations (Table 1, Methods), principally because they were predicted using both MAQ and SSAHA2 but also because they generate consistently low read coverage over an extended region. Also, three (chr04, 05, 07) were previously identified in the progenitor clone AS-30CQ or in a related clone AS-15MF [25,26]. One of these was previously confirmed by dideoxy-sequencing as an intergenic 34 bp deletion on chr07. Another was a potential large deletion (>1 kb) on chromosome 5 located within a gene coding for a non-syntenic (*ie*, its chromosomal location is not conserved across malaria species relative to surrounding genes) S-antigen (PCHAS_051910-20). The third was a deletion in non-coding sequence at the right hand end of chromosome 4, in a region previously defined to be the site of deletion and translocated copy of a large duplicated chr12 fragment containing *mdr1* in the related clone AS-15MF [27]. The remaining high confidence large deletion corresponds to a potential ~23 kb deletion on an unassigned contig (‘bin’ contig11844), not predicted in the progenitor clone AS-30CQ, spanning several non-syntenic genes (PCHAS_000700 to PCHAS_000760). The regions corresponding to deletions on chr05 and in contig 11844 also contain an example of a SNP ‘proxy for deletion’ (see section above). Verification of the three deletions (in chr04, chr05 and contig 11844) by di-deoxy sequencing was not possible due either to their size or position or both. For the other 10 larger deletion candidates, six are intergenic and four are predicted to interrupt *P. chabaudi*-specific genes, three mapping to the extreme ends of chr13 (two) and chr14, and one to an unassigned contig (see Additional file 5).

It is worth noting that the frequency of indels in the chicken and human genomes (e.g. [46,47]) is less than the SNP frequency; confirmed in next generation short-sequencing analysis [48]. If applicable to *Plasmodium* spp, the number of indels will be less than the number of point mutations. Since the number of point mutations in AS-ART (relative to AS-sens) is likely to be eight, the four high-confidence deletions detailed above may represent the full complement of indels. Only one (in contig 11844) appears in AS-ART, relative to AS-30CQ.

For the seven potential CNVs, identified by higher read comparative coverage (range 1.5 – 3.1), they extend over very small regions (205–282 bp) and lie outwith coding regions of genes (see Additional file 6). In other clones of the *P. chabaudi* AS lineage, previously validated large-fragment CNVs (involving *mdr*1) were identified using a similar comparative read-coverage analysis ([27] and data not shown). In the present case, the seven potential CNVs are unlikely to represent gene duplication/amplification events, simply reflecting natural variation of read coverage.

It is likely that there is only one high-confidence deletion (contig 11844) arising in AS-ART relative to AS-30CQ. The likelihood of other indels or CNVs arising in AS-ART is considered to be low and, in any case, unlikely to confer artemisinin resistance phenotype 2.

### Conservation and structure of AP2 μ-chain

**Figure 3:**
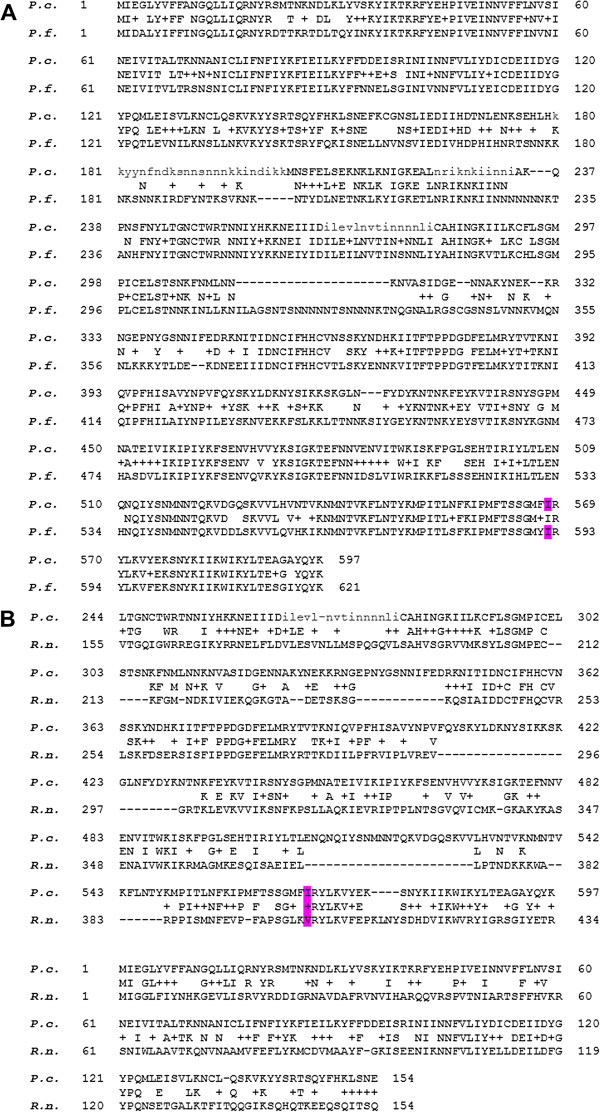
**Conservation of AP2 μ-chain amino acid sequence.** Conservation between *P. chabaudi* (PCHAS_143590) sequence and **A**) *P. falciparum* (PF3D7_1218300) or (**B**) rat (**P84092 **UniProtKB/Swiss-Prot) sequences. The *P. chabaudi* mutated residue I568 and the corresponding *P. falciparum* residue I592 (**A**) and Rat V401 (**B**) are highlighted (purple). Note *Plasmodium* spp.-specific sequence (~90 residues, relative to rat) lying between positions 154–244 (*P. chabaudi*).

An alignment of the predicted amino acid sequences of *AP2-μ* of *P. chabaudi* (PCHAS_143590, 597 aa) and its *P. falciparum* orthologue, PF3D7_1218300 (Figure 3A) and other *Plasmodium* spp (data not shown) confirm that this gene is highly conserved in *Plasmodium* spp. Specifically, the I568T mutation appears close to the C-terminus in a particularly highly conserved region. BLAST searches identify AP2 adaptor *μ*-chains from other species such as *R. norvegicus* (435 aa) (Figure 3B) showing high sequence conservation in both the N-terminal domain (aa 1–154, numbering *P. chabaudi* unless otherwise stated) and the C-terminal domain (aa 244–597) which forms two subdomains each composed of a β-sheet. These large regions of β-sheets bind to, and hence select cargo protein during coated pit formation and vesicle formation during endocytosis [45,49].

In addition to the conservation of its amino acid sequence, other sequence and structural features of PCHAS_143590 support its designation as the *μ*-chain of the AP2 adaptor complex and a role in recognition of cargo protein and endocytosis. Residues 155 to 243 comprise *Plasmodium* specific sequence and extend the linker (rat136 – 158) between the *AP2-μ* N-terminal and C-terminal domains. At its C-terminal end, this linker contains T245, homologous to rat T156 that requires phosphorylation for endocytosis *in vitro* and *in vivo*[50]. Also, the *P. chabaudi* protein contains 17 lysine residues (aa 410 – 537) in a region where positively charged patches in rat μ chain interact with phosphatidylinositol bisphosphate (PIP_2_) [51]. Both phosphorylation and PIP_2_ or PIP_3_ binding stabilize a large conformational change that allows cargo signal motifs to access the μ-chain signal recognition site at the membrane surface [52].

BLAST alignments (Figure 3A, 3B) support the designation of *P. falciparum* I592 and *R. norvegicus* V401 as the corresponding residues to *P. chabaudi* I568, suggesting that these residues will have similar positions within the protein structure, and therefore, a corresponding functional significance.

**Figure 4:**
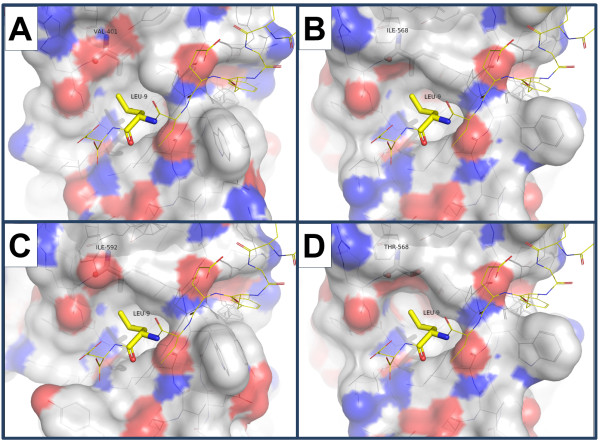
**I568T mutation in AP2 μ-chain interacts with YXXΦ motif on cargo protein. A**. Rat (P84092) V401 is homologous to *P. chabaudi* AP2 μ-chain I568, and contributes to the hydrophobic pocket that binds L9 residue of bound peptide (DEEYGYECL) in structure 2PR9. **B**. Homology model for *P. chabaudi* wild-type homologue shows similar structure with I568 corresponding to rat V401. **C**. Homology model for *P. falciparum* orthologue shows similar structure, with I592 corresponding to rat V401 and *P. chabaudi* I568. **D**. Homology model for *P. chabaudi* mutant - 568T is predicted to reduce the hydrophobic character of the binding pocket in which peptide L9 binds. Note increased polar character associated with threonine hydroxyl group in **D** relative to **B**.

X-ray structures for both the *μ*-chain alone or the adaptor complex cocrystallised with peptides containing YXXΦ (where Φ represents a hydrophobic residue such as F, I, V, L or M) recognition motifs on the target (cargo) ligands [53] include a rat *μ*-chain structure (pdb 2PR9), showing that the V401 residue of the rat *μ*-chain is adjacent to the L9 Φ residue (L, lysine) of the bound DEEYGYECL peptide and, along with rat L173, L175, L404 and V422, forms a hydrophobic pocket into which the Φ residue binds (Figure 4A). The high sequence conservation between PCHAS_143590 and the rat AP2 μ-chain (Figure 4B, Figure 3B) suggests that I568 will play a similar role in the *P. chabaudi* AP2 adaptor to that of V401 in the rat homologue. Homology models of the *P. chabaudi* structure were predicted by threading the *P. chabaudi* amino acid sequence into the rat crystal structure, using I-TASSER. The resulting structures indeed show that I568 (*P. chabaudi*, Figure 4B) and I592 (*P. falciparum*, Figure 4C) are predicted to lie in a corresponding position, relative to the Φ residue in the bound peptide. The likely effect of the I568T mutation was investigated in the *P. chabaudi* homology model. The most likely conformation of 568T was predicted by minimising the contact of the side chain oxygen atom in 568T to nearby hydrophobic residues. This results in the oxygen pointing towards the solvent, hence forming part of the Φ-binding pocket (Figure 4D). The effect of the 568T hydroxyl group is therefore to add some hydrophilic character to the hydrophobic pocket into which the Φ residue fits. Similar results were obtained for the *P. falciparum* μ2 homology model (data not shown). The I568T mutation is predicted to moderate but not abrogate the binding of the μ subunit to the YXXΦ recognition motif present in the cargo protein. In any case, at least in yeast, an AP-2 *μ*-chain knock-out is not lethal [54] and clathrin may function independently of the adaptor [55].

### Polymorphisms in *Plasmodium falciparum ap2-μ* gene

Because the only confirmed point mutation arising in *P. chabaudi* AS-ART along with ART resistance phenotype 2 lies in the *AP2-μ* gene, the polymorphisms in the *P. falciparum* orthologue (PF3D7_1218300) were investigated. The DNA sequence analysis of PF3D7_1218300 in 24 samples (see Additional file 2) from Brazil, São Tomé and Rwanda revealed a total of 12 independent polymorphic sites, consisting of nine SNPs and three indels. Seven SNPs and all the insertions were novel genetic variants (relative to existing information in PlasmoDB, Table 2) and unrelated to each other. A polymorphism similar to *P. chabaudi* I568T was not detected in any of the isolates inspected.

**Table 2:** **Genetic polymorphisms identified in the *pfap2-μ *gene (PF3D7_1218300)**

**Nucleotide**	**Reference sequence (3D7) variant**	**Codon, variant**	**Amino acid substitution**	**Location**
300	ATA	ATC	I100syn	Rwanda
381	GTG	GTT*/GTC	V127syn	Brazil
**437**	AGA	AAA	**R146K**	Rwanda
**479**	AGT	AAT	**S160N**	Rwanda, STP
486	ATT	ATC	I162syn	Rwanda
489	GAA	GAG*	E163syn	Rwanda, STP
**596**	AAA	ACA	**K199T**	Rwanda
**699**	AAT	AAG	**N233K**	Rwanda
**1311**	TTC	TTA	**F437L**	Rwanda
**Insertions**				
		**Codon insertion**	**Amino acid insertion**	
**699**	AAT	AATAAT	**233 + N**	STP
**972**	AAT	AATAAT	**324 + N**	Rwanda, STP
**987**	AAT	AATAAT	**329 + N**	STP

Polymorphic bases are underlined. * - SNP previously identified and recorded in PlasmoDB. Non-synonymous SNPs are shown with amino acid substitutions (bold). STP, Sao Tome and Principe.

Five of the nine SNPs were non-synonymous (aa 146, 160, 199, 233 and 437) while four were synonymous (Table 2). The non-synonymous mutations R146K, S160N and F437L occur within regions which appear to be well conserved in *Plasmodium* spp of the N-terminal domain of *AP2-μ* (Figure 3A), although residues in these regions are not well-conserved relative to the rat homologue (Figure 3A, B). The non-synonymous SNPs, K199T and N233K are situated in unconserved regions. The three indels were three-nucleotide insertions of an AAT codon (asparagine) found adjacent to codon 233, 324 or 329 in asparagine-rich sequence (Table 2) not conserved in the *P. chabaudi* orthologue. Amino acids 324 and 329 are located on a *P. falciparum*-specific region relative to both *P. chabaudi* and the rat homologue (Figure 3A, B).

## Discussion

### Increased artemisinin resistance phenotype

An ART resistance phenotype (phenotype 1) in *P. chabaudi* was previously described and the underlying genetic mechanism (mutations in *ubp1*) identified [25]. That phenotype had been experimentally evolved in AS-30CQ under chloroquine selection and without any exposure to ART. Subsequently AS-ART was evolved from AS-30CQ by ART selection [29]. Here, an increased artemisinin resistance (phenotype 2) in AS-ART relative to AS-30CQ is characterized. The increased resistance is apparent at 200 mg ART kg^-1^ d^-1^ (inoculation of 10^6^ parasites, three-day drug treatment). Interestingly a five-day treatment using a higher inoculum (10^7^ parasites) also differentiated AS-ART from AS-30CQ. These data are consistent with the hypothesis that quiescence or dormancy responses may underlie survival of artemisinin treatment [56,57].

### Genetic basis of increased artemisinin resistance

Illumina whole-genome sequencing reveals a single point mutation arising between AS-30CQ and AS-ART. This mutation encodes an I568T substitution in the μ subunit of the AP2 adaptor complex (PCHAS_143590). The number of false negatives due to insufficient coverage is estimated to be less than one/genome, as previously discussed [25,26] and given the minimum threshold for point mutation detection (three reads), the proportion of the genome covered by less than three reads (1.67%, see Additional file 4) and the accurate identification of simulated mutations (random alterations in reference sequence) [25]. The frequency of a false negative arising from incorrect read alignment is minimized because the re-sequenced and reference genomes are largely identical, and because optimal hashing-table algorithms were used. Importantly the likely appearance of only one point mutation is consistent with previous genome re-sequencing studies [26] that reveal a consistent set of a small number of point mutations, most of which confer drug-resistance phenotypes in other drug-resistant clones of the *P. chabaudi* AS lineage (Table 1) [25-28]. For example, four or five out of seven single nucleotide substitutions between AS-sens and AS-30CQ confer resistance to pyrimethamine, chloroquine and artemisinin phenotype 1 [25,26]. This is consistent with similar studies using other parasites; for example, only four non-synonymous mutations arose in each of two miltefosine-selected *Leishmania major* genomes [58].

Although the possibility that increased ART-R is conferred by unidentified point mutations in the small fraction of the unassembled genome cannot be excluded, regions of low read coverage, the indel identified in AS-ART, or further unidentified indels, suggest that the mutation I568T in *AP2-μ* on chr14 is the principal determinant of higher-level artemisinin resistance (ART-R phenotype 2) in AS-ART which also bears the *ubp1* mutation [25] but no *mdr1* amplification [27]. This hypothesis should be validated by transfection experiments.

Other results are consistent with this hypothesis. A previous study suggested that a *locus* on chr14 contributes to artemisinin responses in genetic selection experiments using AS-ART [23]. Also, two recent independent reports show evidence of the involvement of adaptor proteins in drug resistance phenotypes in other human parasites. A genome-wide RNAi target sequencing approach revealed *Trypanosoma brucei* parasites with a knockdown of one of each of the four AP1 adaptor subunit genes (as well as lysosomal proteases, vacuolar protein sorting factors, etc.) of the endosomal system in suramin-treated populations [59]. In a similar experimental paradigm to that described here, an *in vitro* miltefosine-resistant *L. major* line bore a D762 mutation in the α-subunit of an adaptor protein and a different mutation in the *Leishmania infantum* orthologue [58]. The probability that mutations in adaptor subunits confer resistance to three structurally and functionally unrelated drugs (suramin, miltefosine and artemisinin) in three different parasites (*Trypanosoma, Leishmania* and *Plasmodium*) suggests that a conserved aspect of drug treatment and response is targeted by these mutations.

### Clathrin-mediated endocytosis - insights regarding ART action and resistance

The AP2 adaptor complex is a heterotetramer (α, β, μ, σ) that selects and recruits other factors, including membrane protein (cargo) and intracellular clathrin, mediating clathrin-mediated endocytosis (CME) [45]. CME is involved in the internalization of extracellular molecules and ligands (e g, low density lipoprotein, transferrin, growth factors, antibodies and bacterial toxins), the remodelling of the plasma membrane (removal of variable antigen), and membrane protein trafficking and vesicular sorting through the endosomal system and lysosomes [49]. In *P. falciparum*, these cellular processes are being studied particularly regarding digestive vacuole genesis, haemoglobin uptake and digestion [60], the role of (poly)phosphorylated phosphatidyl inositol [61], the action of aminoquinolines and artemisinins [6,7,62-64] and the trafficking of a critical determinant of chloroquine resistance (the membrane protein, *crt*) [65]. The participation of clathrin (as coated vesicles) in endocytosis and its relationship to the formation of cystosomes has not been fully clarified and, in any case, the mechanism would require the production of vesicles with double membranes (parasitophorous vacuole and parasite membranes). Nevertheless, it is possible that some elements of CME have been recruited to the haemoglobin uptake mechanism. The existence of *Plasmodium*-specific additional sequence between the N- and C-terminal domains of the *AP2-μ* chain may reflect non-canonical functional attributes of the AP2 adaptor complex.

The I568T mutation in the AP2 μ-chain locates to a residue forming a hydrophobic pocket into which the Φ residue of the YXXΦ recognition motif on the cargo proteins binds. This suggests that modulation of cargo trafficking may be the key event in this resistance pathway. The I568T mutation in *AP2-μ* may reduce its binding to membrane cargo and mediates artemisinin resistance phenotype 2 by reducing endocytosis or the recycling or trafficking of one or more membrane proteins. Previous work has shown that fluorescent artemisinin derivatives concentrate in neutral lipid bodies close to the digestive vacuole [5], suggesting that drug may be taken up by endocytosis. Artemisinin interacts with the endocytic and endosomal pathway. For example, it inhibits endocytosis [62] and haemoglobin uptake [7], resulting in the disruption of the DV membrane [6] and some derivatives may increase the accumulation of endocytic vesicles [6]. Also, artemisinin activity is dependent on haemoglobin digestion [7]. A reduction in haemoglobin uptake or digestion may therefore constitute a strategy for surviving exposures to short-lived artemisinins. The I568T mutation might therefore reduce the rate of haemoglobin uptake and hence the activation or action of artemisinin. Indeed, the reduced growth of AS-ART in relation to its sensitive progenitor AS-30CQ in absence of treatment (Figure 2A), indirectly supports this notion. The involvement of adaptor proteins in resistance to suramin, miltefosine and artemisinin in *Trypanosoma*, *Leishmania*[66,67] and *Plasmodium* however, may suggest a rather general resistance mechanism involving, for example, the reduction of drug uptake through endocytosis.

A V2728F mutation in *ubp1* was previously identified as the critical determinant of artemisinin resistance phenotype 1 in AS-30CQ [25], predicting that this mutation would decrease de-ubiquitination. The ubiquitination of membrane receptors and proteins may induce clathrin-independent (caveolae) endocytosis [68]. Therefore, the *ubp1* and *AP2-μ* mutations may both modulate endocytosis, possibly changing the balance of endocytosis toward a clathrin-independent pathway. Interestingly, chloroquine is also known to interact with endocytosis, vesicular transport, haemoglobin digestion and actin and DV dynamics [62-64,69] and the V2728F *ubp1* mutation has been shown to confer high-level CQ-resistance [26]. This artemisinin- and chloroquine-cross resistance is therefore consistent with the suggestion that endocytic pathways are involved in the mode of action of both of these drugs and the resistance mechanisms in *P. chabaudi*.

The present study therefore suggests that experimental studies on the molecular interactions between ubiquitination pathways, endocytosis and artemisinin uptake and sensitivity may provide important insights regarding artemisinin action and resistance mechanisms.

### Field studies of *Plasmodium falciparum*

12 genetic polymorphisms (nine SNPs and three insertions) were identified in the *P. falciparum* orthologue (PF3D7_1218300) in field samples from Rwanda, São Tomé and Brazil. Further studies will therefore be required to evaluate the association between PF3D7_1218300 polymorphisms and artemisinin responses. Association studies in locations in Southeast Asia where ART-R has been reported would be of particular interest. A recent linkage analysis of a *P. falciparum* genetic cross (HB3 × Dd2) revealed three *loci* linked to *in vitro* artemisinin responses; on chr05, chr12 and chr13 [24]. Marker C12M63 was the most highly linked marker on chr12. However, this marker maps over 1 Mbase downstream from PF3D7_1218300 suggesting that a different gene was involved in that study. A genome-wide analysis of genetic diversity in *P. falciparum* parasites from Asia, with different artemisinin responses mapped a possible genetic determinant to a region of chr13 [21]. However, no SNPs were identified in these genes that could be associated with the *in vivo* parasite’s responses to ART. Recently however, the genotyping of *P. falciparum* isolates from Asia established associations between four SNPs (on chromosomes 10, 13 and 14) and both recent positive selection and parasite clearance phenotypes after artemisinin treatment [70]. However, the genes and regions where these SNPs lie are not linked to the *P. falciparum* orthologue of the AP2 μ-chain on chromosome 12, suggesting a different gene to that identified here is involved

## Conclusions

An increased artemisinin resistance phenotype in a lineage of *P. chabaudi* drug-resistant parasites was identified. Genome-wide re-sequencing identifies a single point mutation in a critical part of the AP2 adaptor μ-chain. This mutation is predicted to increase artemisinin resistance by modulating clathrin-mediated endocytosis. Analysis of the orthologous gene in *P. falciparum* field isolates demonstrates existing genetic variation in the human malaria parasite.

Lastly, the possibility was considered that mutations in a different gene(s) may play a role in responses to artemisinin derivatives in *P. falciparum*. However, further than assigning particular mutations to ART resistance phenotypes, it is suggested that a potentially novel biological pathway through which artemisinin resistance may occur may be involved.

## Competing interests

The authors declare that they have no competing interests.

## Authors’ contributions

GH genotyped field samples. AM performed whole genome analysis. LR, KM, RF collected phenotype data. DH and PH modelled 3D structures. LR, KM, SB validated mutations. Ud’A, HT, CK provided materials. PC and PH supervised study, interpreted data, and wrote the paper. All authors read and approved the final manuscript.

## Supplementary Material

Additional file 1 **Primer sequences and PCR reactions for the *pfap2-μ *gene.** Click here for file

Additional file 2 **Summary of the Solexa whole genome re-sequencing performed on clone AS-ART of *Plasmodium chabaudi.*** Click here for file

Additional file 3 AS-ART Genome re-sequencing – validated and low-confidence point mutations. Click here for file

Additional file 4 AS-ART Genome re-sequencing – low probability small indels. Click here for file

Additional file 5 AS-ART Genome re-sequencing – larger indels. Click here for file

Additional file 6 AS-ART Genome re-sequencing – possible CNVs. Click here for file
