# Supplementary material for: Artemisinin resistance in rodent malaria - mutation in the AP2 adaptor μ-chain suggests involvement of endocytosis and membrane protein trafficking
Source: Malar J. 2013 Apr 5;12:118. doi: 10.1186/1475-2875-12-118 (PMC3655824; doi:10.1186/1475-2875-12-118)
Supplement: Additional file 1 — Primer sequences and PCR reactions for the pfap2-μ gene. [file 1475-2875-12-118-S1.docx]

**Additional file 1.** Primer sequences and PCR reactions for the *pfap2-µ* gene

| **Primers** | **Sequence (5’ to 3’)** | **Coordinates**  **(length)** | **PCR Program** | |
| --- | --- | --- | --- | --- |
|  |  | -54 to 524  (578 bp) |  |  |
| **PfClR-1F** | GTTAACACGATTAGCGTCATTTG |  | 94 ºC, 3 min |  |
| **PfClR-2R** | GTCCTATTATGTATATGTGGATC |  | [94°C, 30’; 53°C, 45’; 72°C, 60’] | 35 cycles |
|  |  |  | 72 °C, 10 min |  |
|  |  | 358 to 1199  (841 bp) |  |  |
| **PfClR-3F** | GATATCCACAAACATTAGAAGTG |  | 94 ºC, 3 min |  |
| **PfClR-4R** | CCATCTGGTGGTGTGAAGG |  | [94°C, 30’; 52°C, 45’; 72°C, 60’] | 35 cycles |
|  |  |  | 72 °C, 10 min |  |
|  |  | 1126 to +58 (753 bp) |  |  |
| **PfClR-5F** | GCATATTTCATCATTGTGTTACC |  | 94 ºC, 3 min |  |
| **PfClR-6R** | ACACCCGATTGAACTATTTATAC |  | [94°C, 30’; 53°C, 45’; 72°C, 60’] | 35 cycles |
|  |  |  | 72 °C, 10 min |  |
