# Supplementary material for: Artemisinin resistance in rodent malaria - mutation in the AP2 adaptor μ-chain suggests involvement of endocytosis and membrane protein trafficking
Source: Malar J. 2013 Apr 5;12:118. doi: 10.1186/1475-2875-12-118 (PMC3655824; doi:10.1186/1475-2875-12-118)
Supplement: Additional file 2 — Summary of the Solexa whole genome re-sequencing performed on clone AS-ART of Plasmodium chabaudi. [file 1475-2875-12-118-S2.docx]

| **Clone analysed** | **AS-ART** |
| --- | --- |
| Reference sequence | Sanger (09/2009) |
| Read length | 36 |
| Total Number of Reads | 45.879.892 |
| Mapped Reads (SSAHA2) | 41.198.901 |
| Uniquely Mapped Reads (SSAHA2) | 39.979.351 |
| % Genome covered by >= 10reads | 98,00% |
| % Genome covered by <3 Reads | 1,67% |
| x coverage (SSAHA2) | 84.16X |
| x coverage (MAQ) | 86.18X |

**Additional file 2.** Summary of the Solexa whole genome re-sequencing performed on clone AS-ART of *Plasmodium chabaudi*
