# Supplementary material for: Artemisinin resistance in rodent malaria - mutation in the AP2 adaptor μ-chain suggests involvement of endocytosis and membrane protein trafficking
Source: Malar J. 2013 Apr 5;12:118. doi: 10.1186/1475-2875-12-118 (PMC3655824; doi:10.1186/1475-2875-12-118)
Supplement: Additional file 3 — AS-ART Genome re-sequencing – validated and low-confidence point mutations. [file 1475-2875-12-118-S3.docx]

**Additional file 3. AS-ART Genome re-sequencing – validated and low-confidence point mutations**

| **Chromosome** | **Position** | **Analysis** | **Reference base** | **AS-ART base** | **SSAHA2 Quality score** | **Confirmation of mutation by dideoxy sequencing** | **P. chabaudi Gene ID** | **Mutation** | ***P. falciparum* orthologue** | **Nearest gene ID (P. chabaudi)** |
| --- | --- | --- | --- | --- | --- | --- | --- | --- | --- | --- |
| **1** | 551,969 | SSAHA | G | A | 3 |  | intergenic |  | None | PCHAS_011470-5’ |
| **2** | 216,954 | SSAHA/MAQ | C | A | 99 | YES | PCHAS_020720 | **V2728F** | PF3D7_0104300 |  |
| **3** | 25,017 | SSAHA | G | A | 5 |  | intergenic |  | None | PCHAS_030080-5’ |
| **3** | 70,553 | SSAHA/MAQ | G | T | 99 | YES | PCHAS_030200 | **T707N** | None |  |
| **3** | 474,123 | SSAHA/MAQ | C | A | 99 | YES | PCHAS_031370 | **T719N** | PF3D7_0214800 |  |
| **5** | 684,091 | SSAHA | T | A | 33 |  | PCHAS_051910-20 | non-syno | None |  |
| **7** | 31,783 | SSAHA | T | C | 5 |  | intergenic |  | PF3D7_1033400 | PCHAS_070080-5’ |
| **7** | 72,468 | SSAHA | T | A | 8 |  | PCHAS_070170 | K-N | None |  |
| **7** | 994,546 | SSAHA/MAQ | G | A | 99 | YES | PCHAS_072830 | **S106N** | PF3D7_0417200 |  |
| **10** | 634,932 | SSAHA/MAQ | T | C | 99 | YES | PCHAS_101550 | **Y162H** | PF3D7_1430000 |  |
| **10** | 1,625,436 | SSAHA | C | T | 7 |  | intergenic |  | None | 5’-PCHAS_104250 |
| **11** | 996,332 | SSAHA/MAQ | G | T | 99 | YES | PCHAS_112780 | **A173E** | PF3D7_0629500 |  |
| **12** | 875 | SSAHA | A | G | 12 |  | PCHAS_120010 | nonsyn | None |  |
| **14** | 936,945 | SSAHA/MAQ | T | G | 92 | YES | intergenic |  | PF3D7_0813400 | 5’-PCHAS_142600 |
| **14** | 1,270,184 | SSAHA/MAQ | T | C | 99 | YES | PCHAS_143590 | **I568T** | PF3D7_1218300 |  |
| **bin** | 5,256 | SSAHA | G | C | 13 |  | PCHAS_000020 | A-G | None |  |
| **bin** | 106,638 | SSAHA | A | C | 3 |  | PCHAS_000260 | N-T | None |  |
| **bin** | 177,899 | SSAHA | A | G | 2 |  | PCHAS_000480 | Y-C | None |  |
| **bin** | 270,747 | SSAHA | T | A | 31 |  | intergenic |  | None | PCHAS_000730-5’ |

Summary of all point mutations proposed prior to stringent analysis and filtering (see text) in AS-ART. Those confirmed by di-deoxy sequencing (green) are indicated. Low probability mutations (orange, see Results) are indicated. For intergenic SNPs, the nearest *P. chabaudi* gene is indicated, with indication as to whether it lies to the left or right of 5’ or 3’ end of gene. For example, 5’ - PCHAS_011470 indicates that the mutation is found to the left (upstream) of the 5’ end of PCHAS_011470. For 11 low-confidence (orange) point mutations, 6 lie within predicted genes (chr05, chr07, chr12 and bin (3)). Two of these (chr05 and chr12) are non-synonymous. Candidate on chr05 is considered as ‘proxy for deletion’ (see main text).
