# Supplementary material for: Artemisinin resistance in rodent malaria - mutation in the AP2 adaptor μ-chain suggests involvement of endocytosis and membrane protein trafficking
Source: Malar J. 2013 Apr 5;12:118. doi: 10.1186/1475-2875-12-118 (PMC3655824; doi:10.1186/1475-2875-12-118)
Supplement: Additional file 4 — AS-ART Genome re-sequencing – low probability small indels. [file 1475-2875-12-118-S4.docx]

| **Chromosome** | **Insertion or Deletion** | **Analysis** | **Nucleotide start** | **Nucleotide finish** | **Size of region** | **Small indel quality index** | **Dideoxy-sequencing validation** | ***P. chabaudi* gene ID** | **Nearest *P. chabaudi* gene ID** |
| --- | --- | --- | --- | --- | --- | --- | --- | --- | --- |
|  |  |  |  |  |  |  |  |  |  |
| **1** | Insertion | SSAHA | 203,105 | 203,105 | 1 | 22/38 |  | intergenic | 5-PCHAS_010530 |
| **2** | Insertion | SSAHA | 32 | 32 | 1 | 5/10 |  | intergenic | 3-PCHAS_020010 |
| **4** | Deletion | SSAHA | 104,874 | 104,874 | 1 | 33/49 |  | intergenic | PCHAS_040280-5 |
| **5** | Deletion | SSAHA | 281,711 | 281,711 | 1 | 3/5 |  | intergenic | 3-PCHAS_050670 |
| **5** | Deletion | SSAHA | 716,257 | 716,257 | 1 | 23/38 |  | intergenic | PCHAS_052010-5 |
| **6** | Insertion | SSAHA | 430,538 | 430,538 | 1 | 110/118 | NO | PCHAS_061120 |  |
| **6** | Deletion | SSAHA | 678,850 | 678,851 | 2 | 3/6 |  | intergenic | 3-PCHAS_061760 |
| **7** | Deletion | SSAHA | 779,270 | 779,270 | 1 | 314/200 |  | PCHAS_072120 |  |
| **7** | Deletion | SSAHA | 910,035 | 910,035 | 1 | 7/9 |  | intergenic | PCHAS_072520-5 |
| **7** | Insertion | SSAHA | 910,341 | 910,341 | 1 | 9/12 |  | intergenic | 3-PCHAS_072530 |
| **8** | Insertion | SSAHA | 854,448 | 854,448 | 1 | 22/35 | NO | PCHAS_082240 |  |
| **9** | Deletion | SSAHA | 1,113,660 | 1,113,660 | 1 | 62/82 |  | PCHAS_093250 |  |
| **10** | Deletion | SSAHA | 290,661 | 290,661 | 1 | 81/84 | NO | intergenic | 3-PCHAS_100680 |
| **10** | Deletion | SSAHA | 719,577 | 719,577 | 1 | 6/8 |  | intergenic | PCHAS_101800-3 |
| **10** | Deletion | SSAHA | 908,717 | 908,718 | 2 | 118/155 | NO | PCHAS_102390 |  |
| **10** | Insertion | SSAHA | 1,625,438 | 1,625,438 | 1 | 3 / 4 |  | intergenic | 5-PCHAS_104250 |
| **11** | Insertion | SSAHA | 694,180 | 694,180 | 1 | 314/452 |  | intergenic | 3-PCHAS_111970 |
| **11** | Deletion | SSAHA | 760,507 | 760,507 | 1 | 47/62 | NO | PCHAS_112150 |  |
| **11** | Deletion | SSAHA | 836,567 | 836,567 | 1 | 54/80 |  | PCHAS_112370 |  |
| **13** | Deletion | SSAHA | 1,792,799 | 1,792,799 | 1 | 7/13 |  | intergenic | 3-PCHAS_134780 |
| **14** | Insertion | SSAHA | 9,124 | 9,124 | 1 | 3/3 |  | intergenic | 3-PCHAS_140040 |
| **14** | Deletion | SSAHA | 54,440 | 54,440 | 1 | 3/6 |  | intergenic | PCHAS_140140-3 |
| **14** | Insertion | SSAHA | 82,779 | 82,779 | 1 | 3/3 |  | intergenic | 5-PCHAS_140200 |
| **14** | Deletion | SSAHA | 347,257 | 347,257 | 1 | 39/58 |  | PCHAS_140880 |  |
| **14** | Deletion | SSAHA | 926,444 | 926,444 | 1 | 20/32 |  | PCHAS_142560 |  |
| **14** | Insertion | SSAHA | 1,415,664 | 1,415,664 | 1 | 4/7 |  | intergenic | PCHAS_143940-5 |
| **bin** | Deletion | SSAHA | 352,973 | 352,973 | 1 | 161/166 |  | PCHAS_000930 |  |

**Additional file 4. AS-ART Genome re-sequencing – low probability small indels**

Summary of all small indels predicted (see text) in AS-ART. Those rejected by di-deoxy sequencing are indicated (red). Other small indels (orange) are expected to represent false positives (see text). Quality scores indicate no of reads calling indel/total reads. For intergenic small indels, the nearest *P. chabaudi* gene is indicated, with indication as to whether it lies to the left or right of 5’ or 3’ end of gene. For example, 5’ - PCHAS_010530 indicates that the mutation is found to the left (upstream) of the 5’ end of that gene.
