# Supplementary material for: Artemisinin resistance in rodent malaria - mutation in the AP2 adaptor μ-chain suggests involvement of endocytosis and membrane protein trafficking
Source: Malar J. 2013 Apr 5;12:118. doi: 10.1186/1475-2875-12-118 (PMC3655824; doi:10.1186/1475-2875-12-118)
Supplement: Additional file 5 — AS-ART Genome re-sequencing – larger indels. [file 1475-2875-12-118-S5.docx]

**Additional file 5. AS-ART Genome re-sequencing – larger indels**

| **chromosome** |  | **Analysis** | **Nucleotide start** | **Nucleotide finish** | **Size of region** | **comparative coverage** | **Dideoxy-sequencing validation** | ***P. chabaudi* gene ID** | **Nearest *P. chabaudi* gene ID** |
| --- | --- | --- | --- | --- | --- | --- | --- | --- | --- |
|  |  |  |  |  |  |  |  |  |  |
| **1** | indel | SSAHA | 127 | 149 | 23 | 0.17 |  | intergenic |  |
| **3** | indel | SSAHA | 62 | 207 | 146 | 0.12 |  | intergenic | 5-PCHAS_030010 |
| **4** | indel | SSAHA/MAQ | 793,940 | 793,988 | 49 | 0.16 | tbc | intergenic | PCHAS_042080-5 |
| **5** | indel | SSAHA | 544,355 | 544,403 | 49 | 0.24 |  | intergenic | PCHAS_051440-5 |
| **5** | indel | SSAHA/MAQ | 683,722 | 684,999 | 1,278 | 0.21 | tbc | PCHAS_051910-20 |  |
| **7** | indel | SSAHA | 56,889 | 56,899 | 11 | 0.11 |  | intergenic | PCHAS_070140-5 |
| **7** | indel | SSAHA/MAQ | 876,902 | 876,929 | 28 | 0.18 | 34bp deletion | intergenic | PCHAS_072420-3 |
| **13** | indel | SSAHA | 36,490 | 36,500 | 11 | 0.17 |  | PCHAS_130090 |  |
| **13** | indel | SSAHA | 1,598,774 | 1,598,789 | 16 | 0.22 |  | PCHAS_134220 |  |
| **14** | indel | SSAHA | 943,900 | 943,923 | 24 | 0.21 |  | intergenic | 5-PCHAS_142620 |
| **14** | indel | SSAHA | 2,277,029 | 2,277,055 | 27 | 0.24 |  | PCHAS_146230 |  |
| **bin** | indel | SSAHA | 116,319 | 117,071 | 753 | 0.19 |  | intergenic | 3-PCHAS_000290 |
| **bin** | indel | SSAHA/MAQ | 261,129 | 284,496 | 23,368 | 0.02 | tbc | PCHAS_000700-760 |  |
| **bin** | indel | SSAHA | 300,040 | 300,050 | 11 | 0.19 |  | PCHAS_000790 |  |

Regions of low comparative coverage (see Methods) were identified. 4 higher probability larger indels (see text) are indicated (yellow, not validated or green, validated). Indels of low probability are indicated (orange). For intergenic indels, the nearest *P. chabaudi* gene is indicated, with indication as to whether it lies to the left or right of 5’ or 3’ end of gene. For example, 5’ - PCHAS_030010 indicates that the mutation is found to the left (upstream) of the 5’ end of that gene.
