# Supplementary material for: Artemisinin resistance in rodent malaria - mutation in the AP2 adaptor μ-chain suggests involvement of endocytosis and membrane protein trafficking
Source: Malar J. 2013 Apr 5;12:118. doi: 10.1186/1475-2875-12-118 (PMC3655824; doi:10.1186/1475-2875-12-118)
Supplement: Additional file 6 — AS-ART Genome re-sequencing – possible CNVs. [file 1475-2875-12-118-S6.docx]

**Additional file 6. AS-ART Genome re-sequencing – possible CNVs**

| **chromosome** |  | **Analysis** | **Nucleotide start** | **Nucleotide finish** | **Size of region** | **comparative coverage** | **Dideoxy-sequencing validation** | ***P. chabaudi* gene ID** | **Nearest *P. chabaudi* gene ID** |
| --- | --- | --- | --- | --- | --- | --- | --- | --- | --- |
|  |  |  |  |  |  |  |  |  |  |
| **3** | CNV | SSAHA | 349,895 | 350,122 | 228 | 2.90 |  | intergenic | 5-PCHAS_031010 |
| **6** | CNV | SSAHA | 678,733 | 678,961 | 229 | 1.50 |  | intergenic | 3-PCHAS_061760 |
| **7** | CNV | SSAHA | 677,772 | 678,012 | 241 | 2.40 |  | intergenic | PCHAS_071850-5 |
| **8** | CNV | SSAHA | 814,443 | 814,647 | 205 | 2.50 |  | intergenic | PCHAS_082110-5 |
| **12** | CNV | SSAHA | 926,886 | 927,167 | 282 | 2.80 |  | intergenic | 5-PCHAS_122590 |
| **14** | CNV | SSAHA | 1,595,074 | 1,595,327 | 254 | 1.60 |  | intergenic | 3-PCHAS_144430 |
| **14** | CNV | SSAHA | 1,758,897 | 1,759,122 | 226 | 3.10 |  | intergenic | 3-PCHAS_144820 |

Regions of high comparative coverage (see Methods) was used to identify potential CNVs. These regions are all considered low probability (orange, see Results). For intergenic CNVs, the nearest *P. chabaudi* gene is indicated, with indication as to whether it lies to the left or right of 5’ or 3’ end of gene. For example, 5’ - PCHAS_031010 indicates that the mutation is found to the left (upstream) of the 5’ end of that gene.
